# Population aging, technological innovation and industrial differentiation

**DOI:** 10.3389/fpubh.2025.1502713

**Published:** 2025-01-27

**Authors:** Chunhua Li, Zhangqing Chen, Wangchun Wu, Bin Gao, Lingfeng Zou

**Affiliations:** ^1^School of Economics, Guangxi University for Nationalities, Nanning, China; ^2^Digital Economy and Population Development Research Center, Guangxi University for Nationalities, Nanning, China

**Keywords:** technological innovation, industrial differentiation, moderating effect, industrialization, inter-regional differences

## Abstract

As China’s aging population deepens and its pace accelerates, it is particularly crucial to rely on technological innovation to drive industrial differentiation. Is there a connection between population aging, technological innovation, and industrial differentiation? Does technological innovation have a moderating effect? Based on the panel data of 31 provinces in China from 2006 to 2022, this paper constructs the entropy index to measure the overall industrial differentiation and tertiary industrial differentiation in China, and subsequently investigates the relationship among the three using the two-way fixed effect model. The results indicate that population aging has a significant positive impact on the overall industrial differentiation in China, with a regression coefficient of 1.1025. Technological innovation plays a positive moderating role, with an interaction coefficient of 0.3489. The effects of population aging on the differentiation of the three industries differ: the regression coefficient for the primary industry is −0.6437, which is significantly negative; for the secondary industry, the regression coefficient is 0.9252, which is statistically insignificant; and for the tertiary industry, the regression coefficient is 0.1539, which is significantly positive. The government should encourage enterprises to invest in technology research and development through tax cuts and subsidies, and enterprises should absorb high-quality talents, carry out intelligent transformation of traditional industries of enterprises, and improve their competitiveness.

## Introduction

Population aging is a common phenomenon faced by many countries or regions in the world at present, but China’s population aging degree is deeper and the development speed is faster. According to the data of the Population Division of the United Nations, China’s population aging started in 1969 and continued to age from 1969 to 2050, with the proportion of the older adult aged 60 and above in the total population increasing from 6.1% in 1969 to 38.8% in mid-2050. At that time, the aging degree of China’s population will not only be much higher than the average level of developing countries (20.1%), but also higher than the average level of developed countries (34.3%), which is known as the “vanguard of aging” (1).

The deep and rapid aging of China’s population has brought complex and profound challenges to economic development. For the society as a whole, the deepening of population aging will lead to the increasing burden of social pension (2) and social inequality (3), but it will also bring new consumer demand (4), such as increasing the demand for smart home services (5) and long-term care services (6), and stimulate the development of tourism for the older adult, education for the older adult and other industries (7). For enterprises, with the decrease of labor population, the employment cost of enterprises increases (8) and the knowledge level decreases (9), While impacting enterprise production efficiency, it also forces enterprises to undertake technological innovation, which becomes the key for enterprises to adapt to an aging society and enhance competitiveness. Technological innovation not only improves production efficiency and reduces costs but also develops new markets and products to meet the needs of the older adult population, thereby promoting industrial structure transformation and industry development (10).

At present, driven by emerging technologies, various fields have achieved rapid development (11–14), especially in the areas of information technology, biotechnology, and intelligent manufacturing, which have provided new tools and ideas to address the challenges posed by aging. As a populous country with a serious aging population, how to use technological innovation to promote industrial development and achieve high-quality development of China’s economy has become one of the important issues urgently needed to be studied in China. Technological innovation in this process is not only a means to solve problems but also a new engine for driving industrial differentiation and economic growth. It can not only improve enterprise production efficiency and reduce costs but also create new market demands and employment opportunities. Especially in an aging society, technological innovation can not only promote the birth of new industries, such as smart home, telemedicine, and personalized older adult care services, but also facilitate the transformation and upgrading of traditional industries by improving production efficiency and reducing costs. This leads to the diversification and differentiation of industries in an aging society.

However, the current academic discussion on this issue mainly focuses on population aging, technological innovation and industrial structure upgrading or industrial transformation, ignoring the relationship between population aging and industrial differentiation. Although both industrial differentiation and industrial structure transformation contain the meaning of “change,” the direction of change of the two are different: industrial differentiation is a kind of industrial fission or qualitative change, resulting in the emergence of new industries (15–18); The upgrading of industrial structure or industrial transformation reflects the adjustment or improvement of the internal structure of the same industry, the improvement of the industry itself, or the transformation and upgrading from the primary industry to the secondary industry or the tertiary industry (19–21). At present, the phenomenon of industrial differentiation brought by the aging population has begun to emerge, such as the application of artificial intelligence robots in the older adult and disabled people (22), anti-aging (23) and the leading of health consumer products caused by breakthroughs in gene editing (24). However, there is a lack of relevant research in this area.

At present, the aging degree of China’s population continues to deepen, will the aging population bring technological innovation, and then promote the differentiation of industries? The main contributions of this paper are as follows: (1) The influence of population aging on China’s overall industrial differentiation and the third industrial differentiation is discussed. (2) The transmission effect of technological innovation as a moderating variable is analyzed.

## Literature review

Population aging has a positive or negative impact on industrial development. Population affects economic activities mainly through production and consumption, and the change of industrial structure is also affected by the two dimensions of supply and demand. When population is a laborer, it has the attribute of production. On the one hand, population aging will lead to the decrease of labor productivity due to the reduction of workers’ physical fitness, which is not conducive to the adjustment of industrial structure (25, 26). On the other hand, it will accelerate the accumulation of human capital of enterprises and “force” enterprises to replace labor with capital and technology to cope with the rising labor cost caused by the reduction of labor force, thus accelerating the adjustment of industrial structure (10, 27). As consumers, the population has consumption attributes, and people of different ages have different consumption demands (28). The aging of the age structure of the population will change its consumption demand, consumption habits and consumption structure (4), such as increasing the demand for smart home services (5) and long-term care services (6). Stimulate the development of industries such as tourism and education for the older adult (7). Additionally, population aging may impact the changes in capital supply. According to the logic of the life cycle hypothesis, the deepening of population aging could lead to a long-term continuous decline in residents’ savings. The reduction in savings rate may restrict the growth of investment, thereby constraining the development of capital-intensive industries.

At present, the academic community has not reached a consensus on the impact of population aging on technological innovation. In terms of the negative impact, population aging has a negative effect on technological innovation by affecting human capital (29), or negatively affects technological innovation by increasing financial burden and reducing effective labor (30). In terms of positive impact, the aging of population leads to the reduction of effective labor supply, and the rise of labor cost “forces” enterprises to replace labor with technology, thus promoting technological innovation (31). Some scholars believe that population aging has a more complex impact on technological innovation, rather than a one-dimensional positive or negative impact. Cai’s research shows that while population aging has a positive impact on technological innovation by improving labor efficiency, it also has a negative impact on technological innovation by reducing the level of human capital investment and crowding out innovation resources (32). Dou et al. found that population aging reduced the number of patent applications, but improved the quality of patent applications, and the positive effect showed an obvious inverted “U” shape (33). Ma et al. ‘s research results reveal that population aging has no significant impact on China’s technological innovation (34).

Many scholars admit that technological innovation is always an important factor in the discussion of many factors affecting industrial differentiation (35–37). The high degree of penetration of scientific and technological progress and knowledge leads to rapid industrial differentiation (28), and the technological innovation brought by the technology diversion of enterprises also plays a decisive role in industrial differentiation (38). Technological innovation creates new products, forms new markets, and then forms new industries (39). The introduction of new technology into the original industry will not only lead to the extension of the industrial value chain, but also differentiate new industries with technological roots (40).

The relationship among population aging, technological innovation and industrial development is complicated. In terms of the production segment, technological innovation can adjust the direction of the impact of population aging on industrial differentiation by compensating for the quantity of labor, enhancing the quality of labor, and increasing the return on capital; in terms of the consumption segment, technological innovation can innovate consumption models to meet the consumption needs of the older adult population, thereby influencing the direction of industrial adjustment. If population aging and technological innovation are regarded as two equally important factors, both of them play a decisive role in the change of industrial structure (41). If the interaction between the two factors is considered, the upgrading of industrial structure will be negatively affected (42). Most scholars study technological innovation as a moderating variable between population aging and industrial structure upgrading, and believe that technological progress has a significant moderating effect (43), and its effect on different regions is heterogeneous (7). In addition, other studies have shown that when population aging is taken as the threshold variable, technological innovation has a positive impact on the transformation of manufacturing industry, which increases first and then decreases (44).

The above research has laid a solid foundation for the writing of this paper. However, the above research still has room for improvement in the following three aspects. First of all, on the research topic of population aging and industrial development, most scholars discuss it from the perspective of industrial structure upgrading, while few scholars study it from the perspective of industrial differentiation and the formation of new industries. Secondly, regarding the research on the relationship between population aging, technological innovation and industrial development, most scholars focus on the discussion of the relationship between the two, and at the same time, the exploration of the relationship between the three is very little. Finally, most current studies study the “industry” of “industrial differentiation” as a whole, and rarely consider the differentiation of the primary, secondary and tertiary industries.

Based on the United Nations’ definition of an aging population, China had already entered an aging society by the year 2000. However, the National Bureau of Statistics revised the classification boundaries of the three major industries with the “Regulations on the Division of Primary, Secondary, and Tertiary Industries” introduced in 2003, leading to changes in statistical methods, and there was a significant amount of missing data before 2006. To ensure consistency in statistical methods and completeness of data, the starting year for this study is set to 2006. Given the macro nature of industrial differentiation and the need for output values of detailed industries in the calculation of industrial differentiation, it is more reasonable to conduct empirical analysis at the provincial level compared to the enterprise or city level. Therefore, the sample interval for this paper’s empirical analysis is set from 2006 to 2022, covering panel data from 31 provinces in China. This study incorporates population aging, technological innovation, and industrial differentiation into a unified analytical framework to clarify the relationship between population aging, technological innovation, and industrial differentiation, exploring whether population aging affects industrial differentiation, the direction and magnitude of its impact, whether technological innovation plays a moderating role, and whether population aging has different impacts on the differentiation of the three major industries, among other questions.

## Variable description and model setting

In this study, the “industrial differentiation” is more abstract, and the “industrial differentiation degree” is generally used to testify. According to the needs of the research, there are two categories of explained variables in this paper: one is the inter-industry differentiation (*R*) as a whole concept; the other is the differentiation of the first, second and third industries as subcategories, that is, the differentiation within each industry, namely *R1*, *R2,* and *R3*.

However, at present, the academic community has not reached a consensus on how to measure industrial differentiation, such as measuring the change of the proportion of different industries (45), measuring the index of industrial structure upgrading (46), and measuring the entropy index (39). As the research content of this paper includes both inter-industry differentiation and intra-industry differentiation, it refers to the practice of scholars such as Zhao et al. and Luo et al. (39, 47), and expresses the degree of industrial differentiation through the added value of output value: if the added value of an industry continues to increase, it indicates that the greater the adjustment range of the industry, the higher the degree of industrial differentiation. If the output value distribution of each industry is balanced, it means that the adjustment range of the industry is small and the degree of industrial differentiation is smaller, while the entropy value has the meaning of average information. The entropy index is constructed to measure the degree of industrial differentiation, and the formation of emerging industries and the change of industrial structure system in the process of industrial differentiation are represented by the distribution of output value. Let *S_i_* be the output value of the *i* industry, *N* is the total number of industries, *P_i_* is the proportion of the output value of the *i* industry in the total industrial output value, namely:


(1) $$P_{i}=\frac{S_{i}}{\overset{N}{\underset{i=1}{\sum }}S_{i}}$$

The entropy index is defined as follows:


(2) $$EI=\overset{N}{\underset{i=1}{\sum }}P_{i}\log\left(1/P_{i}\right)$$

Among them, the larger *EI* is, the more balanced the distribution of industrial output value is, and the smaller the industrial differentiation degree is.

The calculation formula of industrial differentiation degree *R* is as follows:


(3) $$R=\frac{1}{EI}=\frac{1}{EI=\overset{N}{\underset{i=1}{\sum }}P_{i}\log\left(1/P_{i}\right)}$$

Among them, the larger *R* is, the higher the degree of industrial differentiation is; On the contrary, the smaller *R* is, the smaller the degree of industrial differentiation is. The above calculation method can be used to calculate the differentiation degree *R* between industries, *R1* of the primary industry, *R2* of the secondary industry and *R3* of the tertiary industry. It should be noted that the differentiation degree of the primary industry (*R1*) is measured by the total value of agriculture, forestry, animal husbandry and fishery, and the differentiation degree of the secondary industry (*R2*) is calculated by the added value of industry and construction. The differentiation degree of the tertiary industry (*R3*) is calculated from the added value of the wholesale and retail industry, the added value of the transportation, storage and postal industry, the added value of the accommodation and catering industry, the added value of the financial industry, the added value of the real estate industry and other industries.

### Population aging (*old*)

Population aging is one of the important factors leading to industrial differentiation and remodeling. On the one hand, the change of consumption demand of the older adult not only promotes the rise of some emerging industries, but also brings challenges to traditional industries, which affects the differentiation of industrial structure from the demand side; On the other hand, population aging affects the differentiation of industrial structure on the supply side through its impact on the labor market. At present, there are two main indicators to measure population aging: one is the proportion of the older adult population in the total population, and the other is the old-age dependency ratio (equal to the number of older adult population divided by the number of working population). According to the classification standard of population aging by the United Nations, if the proportion of the population aged 65 and above in a country or region exceeds 7%, or the proportion of the population aged 60 and above exceeds 10%, then the country or region has entered the population aging society (48, 49). The paper measures the degree of aging by the proportion of people aged 65 and over in the total population. The higher this ratio, the deeper the level of aging. Additionally, during the robustness test phase, the older adult dependency ratio (*older*) is used to assess the level of population aging.

### Technological innovation (*lnpan*)

Technological innovation is an important driving force of industrial differentiation. It can not only promote the cross-differentiation between emerging industries and industries in the original industrial system, but also reshape the original industry. Moreover, it can also give rise to new industries, enrich the internal organization and structure of industries, increase the added value of products of emerging industries, and expand the scale of emerging industries. The concept of technological innovation is relatively abstract, and scholars have different indicators to measure technological innovation. Most scholars use the input method and output method to measure technological innovation, among which the input method refers to the measurement of R&D investment funds, and the more R&D investment, the higher the level of technological innovation (50); The output method is measured by the number of patents granted, and the more patents granted, the higher the technological innovation (29, 51). However, some literature also pointed out that due to the characteristics of R&D investment activities, such as high failure rate and strong uncertainty, innovation output can more intuitively reflect the innovation level (52). Therefore, based on the research content and considering the availability of data, this paper chooses the output method, namely the logarithm of the number of patent applications granted, to measure the level of technological innovation.

Drawing on the existing relevant literature and combining with the availability of data, this paper chooses the following four control variables.

### Level of economic development (*lngdp*)

It is measured by the logarithm of regional GDP ^[53]^. The level of economic development helps to optimize the industrial structure, thus affecting the pattern of industrial differentiation.

### Level of investment in fixed assets (*inv*)

It is measured by the proportion of the total social fixed asset investment in the regional GDP (53). Fixed asset investment represents the direction of capital allocation, and capital usually flocking to industries with better development prospects.

### Level of financial development (*finance*)

It is measured by the proportion of the added value of the financial industry in the added value of the tertiary industry (54). The development of the financial industry can promote the formation of capital, improve the efficiency of capital allocation, allocate resources to more efficient sectors, and then affect industrial differentiation.

### Population size (*lnpopu*)

It is measured by the logarithm of the resident population at the end of the year (55). The increase of population size will promote the expansion of market demand and the increase of labor force, promote the development of industrial diversification and the emergence of market segments, and lead to industrial differentiation (Table 1).

**Table 1:** Descriptive statistics of main variables.

Variable	Mean value	Standard deviation	Minimum value	Maximum value
R	1.11	0.19	0.92	2.20
R1	1.06	0.20	0.76	1.68
R2	2.34	0.61	1.44	4.59
R3	0.65	0.04	0.59	0.82
Old (%)	0.10	0.03	0.05	0.20
Older (%)	0.14	0.04	0.07	0.29
lnpan	9.66	1.81	4.22	13.81
lngdp	9.49	1.11	5.66	11.77
inv (%)	0.77	0.28	0.20	1.60
finance (%)	12.96	3.94	3.86	26.06
lnpopu	8.11	0.85	5.65	9.45
edu	8.90	1.188	4.16	12.68

Table presents the distribution of various variables, with a total of 527 observations.

Combined with theoretical analysis and the above previous research results, in order to study the impact of population aging on industrial differentiation, this paper sets the benchmark model as follows:


(4) $$\begin{array}{l} \;R_{\mathrm{it}}=\alpha_{\mathrm{it}}+\beta_{1}old_{\mathrm{it}}+\beta_{2}\mathrm{lngd}p_{\mathrm{it}}+\beta_{3}\mathrm{in}v_{\mathrm{it}}\\ \;+\beta_{4}\mathrm{financ}e_{\mathrm{it}}+\beta_{5}\mathrm{lnpop}u_{\mathrm{it}}+\mu_{\mathrm{it}}\; \end{array}$$

Where, *R* is the industrial differentiation index; *old* is the degree of population aging; *lngdp* is the logarithm of regional GDP; *inv* is investment in fixed assets; *finance* is the level of financial development; *μ* is the random disturbance term, *i* represents the *i*th province, and *t* represents the *t*th year.

In order to test the moderating effect of technological innovation, this paper constructs the interaction term model including population aging and technological innovation on the basis of Model (4), as follows:


(5) $$\begin{array}{l} \;R_{\mathrm{it}}=\alpha_{\mathrm{it}}+\beta_{1}old_{\mathrm{it}}+\beta_{2}\mathrm{lnpa}n_{\mathrm{it}}+\beta_{3}\mathrm{oldlnpa}n_{\mathrm{it}}+\beta_{4}\mathrm{lngd}p_{\mathrm{it}}\\ \;+\beta_{5}\mathrm{in}v_{\mathrm{it}}+\beta_{6}\mathrm{financ}e_{\mathrm{it}}+\beta_{7}\mathrm{lnpop}u_{\mathrm{it}}+\mu_{\mathrm{it}}\; \end{array}$$

Among them, lnpan is technological innovation; oldlnpan represents the product of the aging rate and technological innovation.

This paper takes the panel data of 31 provinces in China from 2006 to 2022 as the sample for empirical test. The data are from the China Statistical Yearbook of each corresponding year.

## Variable description and model setting

As panel data can be estimated by using mixed regression, fixed effect and random effect models, the Hausman test between the three models is needed to determine which model is the best. The test results in Table 2 reveal that the fixed effect model should be selected for regression analysis. All empirical analyses in this paper were conducted using Stata 16.0 software.

**Table 2:** Selection of models.

Purpose of inspection	Results of inspection	Conclusion
Mixed model and fixed effects model selection	*F* test that all ui = 0: *F*(30,491) = 171.11 Prob > *F* = 0.0000	The fixed effects model is selected
Mixed model and random effects model selection	chibar2(01) = 2097.85 Prob> chibar2 = 0.0000	The random effects model is chosen
Random effect model and fixed effect model selection	chi2(5) = 110.89 Prob>chi2 = 0.0000	The fixed effects model is selected

Table 2 conducts the Hausman test to determine the panel regression model.

For the regression results of the benchmark model, the results of Model 1 in Table 3 show that at the significance level of 1%, the deepening of population aging has played a role in promoting China’s industrial differentiation. For the control variables such as fixed asset investment level, financial development level and population size, their impacts on industrial differentiation are all significant at the level of 1%. Among them, the level of financial development and population size have a positive impact on industrial differentiation, while the level of fixed asset investment has a negative impact on industrial differentiation. The possible reason is that fixed asset investment is mainly concentrated in industrial industries, which has a greater effect on industry aggregation than differentiation.

**Table 3:** Benchmark regression results.

Variable	Model 1	Model 2
	Fixed effect model	2SLS
Old	1.1025^***^	0.9876^***^
	(0.0220)	(0.3109)
lngdp	0.0220	0.0074
	(0.0195)	(0.0202)
inv	−0.0405^***^	−0.0339^***^
	(0.0114)	(0.0107)
Finance	0.4802^***^	0.5137^***^
	(0.1111)	(0.1078)
lnpopu	0.4699^***^	0.4550^***^
	(0.0462)	(0.0544)
Constant	−3.0263^***^	−1.8372^***^
	(0.3951)	(0.4980)
Individual fixed effects	Yes	Yes
Time fixed effect	Yes	Yes
*R* ^2^	0.5187	0.934
Sample size	527	496

Model 1 presents the regression results using fixed effects, while Model 2 represents the 2SLS model. The figures in brackets in the table are the corresponding standard errors, and *, * * and * * * indicate the significance level of 10, 5 and 1%, respectively. The following table is the same.

It should be noted that there may be endogeneity between population aging and industrial differentiation, and the regression estimation results may be biased. There may be a bidirectional causal relationship between population aging and industrial differentiation. The possible reasons are as follows: on the one hand, with the deepening of population aging, the labor supply and the demand for products will change accordingly, thus affecting industrial differentiation; On the other hand, smart medical care, smart home and other industries brought about by industrial differentiation have improved the quality of life of the older adult and extended the average life span. Referring to the methods of Lan and Cai et al. (56, 57), this paper takes the one-period lagged aging rate as an instrumental variable and uses the two-stage least squares (2SLS) method to solve the possible endogenous problems. The first-stage regression results show that the F-statistic is 337.65 and significant at the level of 1%, indicating that there is no weak instrumental variable; The regression results of the second stage are shown in Model 2 of Table 3, and the regression coefficient of the aging rate on the industrial differentiation is significantly positive at the level of 1%, indicating that after excluding endogeneity, population aging can still significantly promote industrial differentiation.

After adding the moderator variable of technological innovation on the basis of the benchmark regression model in Table 3, the regression results in Table 4 are obtained. The results show that from Model 3 to Model 5, when other variables are controlled, the partial regression coefficients of population aging on industrial differentiation are all positive, and the regression coefficients of the interaction term between population aging and technological innovation in Model 4 and Model 5 are significantly positive, indicating that technological innovation plays a positive role in the process of population aging on industrial differentiation.

**Table 4:** Results of moderating effect.

Variable	Model 3	Model 4	Model 5(2SLS)
Old	1.0023^***^	0.1790	−0.5643
	(0.2196)	(0.2406)	(0.4307)
lnpan	−0.0186^***^	0.0031	0.0055
	(0.0068)	(0.0072)	(0.0074)
oldlnpan		0.3489^***^	0.4343^***^
		(0.0502)	(0.06514)
lngdp	0.0437^**^	0.0323	0.1173
	(0.0209)	(0.0200)	(0.0210)
inv	−0.0414^***^	−0.0496^***^	−0.0499^***^
	(0.0114)	(0.0102)	(0.0106)
Finance	0.5531^***^	0.5482^***^	0.5789^***^
	(0.1136)	(0.1083)	(0.1062)
lnpopu	0.4484^***^	0.3644^***^	0.2852^***^
	(0.0466)	(0.0416)	(0.6244)
Constant	−2.8862^***^	−2.2213^***^	−0.4655
	(0.3958)	(0.3894)	(0.5652)
Individual fixed effects	Yes	Yes	Yes
Time fixed effect	Yes	Yes	Yes
*R* ^2^	0.5261	0.5699	0.9754
Sample size	527	527	496

Model 3 displays the regression results with fixed effects, Model 4 shows the regression results with technological innovation included as a moderating variable, and Model 5 represents the 2SLS model.

There may be several reasons for this regression result. First, the forcing mechanism of population aging on industrial differentiation (58). Although China is still dominated by labor-intensive industries at present, with the increase of the older adult population, labor has gradually become a scarce resource, and the supply of labor is less than the demand, resulting in the rising price of labor, and enterprises have changed from labor advantages to technological advantages for survival and development. That is, population aging will force labor-intensive industries to transform into technology-intensive industries, which will promote the differentiation of industries in the process of industrial transformation. Second, technological innovation has a significant positive impact on industrial differentiation. Technological innovation is an important driver of industrial differentiation, which can not only promote industrial differentiation itself, but also promote industrial differentiation by influencing demand structure, improving labor productivity, changing employment structure, and fostering new industries. Third, the aging population promotes the development of the aging industry. According to the life cycle hypothesis (59), the older adult population has a certain amount of wealth accumulation and more disposable income, so there are more potential consumers in the corresponding older adult consumption market. In addition, the government’s supporting policies for the older adult service industry will promote the development of the older adult industry dominated by the tertiary industry to a certain extent. And then accelerate the differentiation of the tertiary industry.

The impact mechanism of population aging and technological innovation on industrial differentiation has been discussed above. In order to ensure the robustness of the results, this paper conducts robustness tests by means of variable replacement, adding control variables, and sample sub-interval model estimation.

First, the core explanatory variables are replaced, and the old-age dependency ratio is used to replace the proportion of the older adult population aged 65 and above in the total population for robustness test.

Second, existing studies have shown that educational level is also an important factor causing industrial differentiation. Therefore, educational level (*edu*) is added to the benchmark model and the moderating effect model for regression, and the average educational year is used to measure the educational level. The specific calculation formula is as follows: Average years of education = (number of illiterate *1+ number of people with primary school education *6+ number of people with junior high school education *9+ number of people with senior high school and technical secondary school education *12+ number of people with junior college degree or above *16)/total population aged 6 or above.

The regression results are shown in Model 7 and Model 10 in Table 5.

**Table 5:** Robustness test results.

Variable	Benchmark regression			Moderating effect		
	Model 6	Model 7	Model 8	Model 9	Model 10	Model 11
	Variable substitution method	Add control variable	Sample subinterval model estimation	Variable substitution method	Add control variable	Sample subinterval model estimation
Old	—	1.1147^***^	0.5099^**^	—	0.1869	0.0688
		(0.2179)	(0.2286)		(0.2401)	(0.2379)
Older	0.6939^***^	—	—	0.0961	—	—
	(0.1332)			(0.1604)		
lnpan	—	—	—	−0.0010	0.0021	0.0020
				(0.0073)	(0.0072)	(0.0073)
oldlnpan	—	—	—	—	0.3495^***^	0.3261^***^
					(0.0501)	(0.0668)
olderlnpan	—	—	—	0.2007***	—	—
				(0.0352)		
lngdp	0.0240^***^	0.0133	0.0581^***^	0.0258	0.0229	0.0548^**^
	(0.0195)	(0.0204)	(0.0202)	(0.0205)	(0.0207)	(0.0213)
inv	−0.0433^***^	−0.0384^***^	−0.0694^***^	−0.0532^***^	−0.0471^***^	−0.0611^***^
	(0.0114)	(0.0115)	(0.0120)	(0.0111)	(0.0110)	(0.0117)
Finance	0.4851^***^	0.4586^***^	0.3038^***^	0.5484^***^	0.5260^***^	0.3958^***^
	(0.0011)	(0.1121)	(0.1152)	(0.1099)	(0.1088)	(0.1151)
lnpopu	0.4556^***^	0.4739^***^	0.3772^***^	0.3640^***^	0.3679^***^	0.3318^***^
	(0.0444)	(0.0463)	(0.0011)	(0.0452)	(0.0460)	(0.0459)
edu	—	0.0122	—	—	0.0148^*^	—
		(0.0088)			(0.0084)	
Constant	−2.9139^***^	−3.0834^***^	−2.5016^***^	−2.1205^***^	−2.2818^***^	−2.1106^***^
	(0.3762)	(0.3969)	(0.3942)	(0.3833)	(0.3900)	(0.3898)
Individual fixed effects	Yes	Yes	Yes	Yes	Yes	Yes
Time fixed effect	Yes	Yes	Yes	Yes	Yes	Yes
*R* ^2^	0.5202	0.5206	0.5483	0.5574	0.5728	0.5774
Sample size	527	527	434	527	527	434

Models 6–8 are robustness checks for the fixed effects, and Models 9–10 are robustness checks for the moderating effects.

Third, the regression model of sample sub-intervals is estimated. In order to exclude the impact of COVID-19 on the data, the data from 2020 to 2022 are excluded, and the model estimation of the sample sub-interval is re-conducted. The regression results are shown in Model 8 and Model 11 in Table 5.

The positive and negative sign directions of the coefficients of the robustness test results above are the same as those of the benchmark regression and the moderating effect regression results, indicating that the impact of population aging and technological innovation on industrial differentiation obtained above is robust.

The above regression analysis reveals that population aging has a significant promoting effect on industrial differentiation. However, since the evolution and differentiation of industries in history has roughly experienced a process from the primary industry to the secondary industry, and then to the tertiary industry, in order to further understand whether population aging will bring different impacts on the three industries, this paper calculates the differentiation degrees of the primary industry, the secondary industry, and the tertiary industry respectively, and replaces the overall industrial differentiation degree above for regression analysis.

In the regression results in Table 6, it can be seen from Model 12 that the impact of population aging on the differentiation degree of the primary industry is negative and significant at the level of 1%. This may be due to the shortage of agricultural labor supply caused by the aging population, or the fact that most of the older adult population in rural areas still carry out agricultural production according to the working habits and skills of young people, which limits the innovation and development of agricultural production (60), thus inhibiting the differentiation of the primary industry.

**Table 6:** Regression results of population aging and the three industrial differentiation.

Variable	Model 12	Model 13	Model 14
	R1	R2	R3
Old	−0.6437^***^	0.9252	0.1539^**^
	(0.2299)	(0.9746)	(0.0779)
lngdp	−0.0047	0.8680^***^	−0.0193^***^
	(0.0205)	(0.0871)	(0.0070)
inv	0.0086	−0.3540^***^	−0.0227^***^
	(0.0121)	(0.0511)	(0.0041)
Finance	−0.4755^***^	1.7307^***^	−0.3399^***^
	(01172)	(0.4970)	(0.0397)
lnpopu	−0.1045^**^	0.0501	0.1099^***^
	(0.0440)	(0.2068)	(0.0165)
Constant	2.0373^***^	−5.2058^***^	−0.0500
	(0.4169)	(1.7670)	(0.1412)
Individual fixed effects	Yes	Yes	Yes
Time fixed effect	Yes	Yes	Yes
*R* ^2^	0.0868	0.6318	0.6195
Sample size	527	527	527

Models 12 and 13, respectively, represent the impact of population aging on the differentiation degree of the primary, secondary, and tertiary industries.

Model 13 in Table 6 shows that the impact of population aging on the differentiation degree of the secondary industry is not significant at the level of 10%. Possible reasons are as follows. First, changes in the labor supply side (61). With the deepening of population aging, the young labor force is gradually decreasing, which brings the shortage of labor force to the secondary industry and leads to the rise of wage cost, which increases the operating cost of enterprises and is not conducive to the development of enterprises. However, the old labor force also has rich experience and skills, which can bring competitive advantages to technology-intensive enterprises. Second, changes in the market demand side (62). With the increase of the older adult population, their demand for traditional manufacturing products, such as automobiles, textiles and housing, may decrease, thus causing a certain negative impact on the traditional manufacturing industry, but their demand for daily necessities and specific products still exists. Moreover, considering the significant differences among various sectors within the secondary industry, the varying degrees of technological substitutability are an important consideration. For those industries with higher technological content that can be easily upgraded and transformed through automation or intelligent means, they are better able to adapt to the challenges brought by changes in the labor force structure. In contrast, traditional manufacturing processes that rely on manual operations and are difficult to quickly transform are more vulnerable to impacts. These factors work together to make the differentiation degree of population aging on the secondary industry insignificant.

Through Model 14 in Table 6, it can be seen that the impact of population aging on the differentiation degree of the tertiary industry is positive and significant at the level of 5%. There are four possible reasons. First, with the aggravation of population aging, the older adult’s demand for healthy older adult care services is also gradually increasing, which provides more development opportunities for medical care, rehabilitation services and community older adult care services (27). Second, as the older adult population increases, the demand for tourism and leisure services also increases, which provides more market space for services such as tourism, hotels, and culture and entertainment. Third, some older adult people continue to study and participate in various training to meet their own interests and needs while providing for their retirement, which provides more development possibilities for the education and training service industry. Fourth, the older adult population has an increased demand for emerging products such as medical devices, health care products and smart home (63), which will guide the government and enterprises to increase investment in the service industry and scientific and technological innovation, thus promoting the differentiation of the tertiary industry.

## Conclusion and suggestions

Based on the panel data of 31 provinces in China from 2006 to 2022, this paper constructs the entropy number to calculate the industrial differentiation degree, uses the fixed effect model and the moderating effect model to conduct an empirical analysis on the relationship between population aging, technological innovation and industrial differentiation, and conducts endogeneity treatment and robustness test, and obtains two main conclusions.

First, in the benchmark regression analysis, control variables such as economic development level, fixed asset investment, financial development level and population size are added to conduct two-way fixed effect model analysis, and it is found that population aging plays an important role in the whole process of industrial differentiation, with a regression coefficient of 1.1025 and a *p*-value less than 0.01, indicating that the deepening of population aging is conducive to industrial differentiation. Further research finds that technological innovation plays a positive moderating role in the impact of population aging on industrial differentiation, with a moderating coefficient of 0.3489 and a *p*-value less than 0.01, indicating that technological innovation will promote the positive impact of population aging on industrial differentiation.

Secondly, although population aging plays a significant role in promoting the overall industrial differentiation, it has different effects on the differentiation among the three industries, with regression coefficients of −0.6437 (*p*-value <0.01), 0.9252 (*p*-value >0.1), and 0.1539 (p-value <0.05), respectively. This indicates that population aging plays a significantly negative role in the differentiation of the primary industry, not a significant role in the differentiation of the secondary industry, but a significantly positive role in the tertiary industry.

China’s aging degree deepening has been a consensus from all walks of life. With the increase of the older adult population, it will stimulate new consumer demand and change the existing labor supply, which will bring about industrial differentiation and then form new industries. Therefore, all sectors of society, including the government and enterprises, should take a positive attitude to deal with population aging, objectively face the irreversible development trend of population aging, and seize the new development opportunities brought by aging. At the enterprise end, it is necessary to seize the consumer demand of the older adult group, invest in the development of many older adult industries such as education for the older adult, medical care for the older adult, nursing services for the older adult and tourism for the older adult, and accelerate the development of the older adult industry. Specifically, companies can increase investment in technological research and development, particularly in technologies that can improve the quality of life for the older adult; establish cooperation with other businesses, research institutions, and government departments to jointly develop new markets and products; and carry out intelligent transformation of traditional industries to improve production efficiency, reduce costs, and enhance competitiveness. For instance, in the primary industry, such as agriculture, enterprises can introduce smart agricultural technologies to increase production efficiency and crop quality, and reduce dependence on labor. On the government side, it is necessary to give certain preferential support policies to the emerging older adult industries, promote the healthy and orderly development of the older adult industries, and pay attention to planning the development of new industries suitable for the older adult group. Specifically, the government can implement the following measures: provide tax relief and financial subsidies, especially for businesses dedicated to developing products and services for the older adult; strengthen cooperation with educational institutions to cultivate and introduce talent specifically for the older adult industry; and optimize market entry processes while enhancing regulation to ensure the quality of products and services. For example, in the tertiary sector, particularly in the service industry, the government can support the development of technologies such as telemedicine, online education, and smart home devices to meet the special needs of the older adult population, while also creating new employment opportunities.

Technological innovation can directly or indirectly promote industrial differentiation, and at the same time, it also plays a moderating role in the impact of population aging on industrial differentiation. At the level of high-tech innovation, population aging has a significant promoting effect on industrial differentiation. Therefore, technological innovation is of great significance to industrial differentiation. In this regard, the government can encourage enterprises to invest in technology research and development through tax cuts and subsidies, and at the same time create a good education environment and pay attention to the cultivation of talents to enhance the independent innovation ability of enterprises. Through talent introduction and other ways, enterprises can absorb high-quality talents to carry out intelligent transformation of traditional industries of enterprises, adapt to the development of the new era, and improve the competitiveness of enterprises.

This study, while delving into the impact of population aging on industrial differentiation and innovatively introducing artificial intelligence as a moderating variable, discusses the distinct effects of population aging on the three industries, thereby providing a new research perspective and methodology. However, the paper still has deficiencies in the following two aspects. First, regarding the time span of the data, the empirical data selected for this paper ranges from 2006 to 2022, which is relatively short. This is primarily because this time period is a key era where data on technological innovation indicators are relatively accessible; to ensure the completeness of the research data, this time range was chosen. Second, in terms of sample selection, due to the need for output value data of detailed industries in the calculation of industrial differentiation, it is challenging to obtain output value data at the prefecture-level city level, and data on technological innovation at the individual and enterprise micro-levels are also difficult to acquire. Consequently, this paper only uses provincial-level macro data for overall analysis, which makes it difficult to discern the micro-mechanisms between population aging, technological innovation, and industrial differentiation.

Based on the aforementioned limitations, this paper proposes corresponding prospects for future research to address these shortcomings. First, subsequent studies could consider expanding the time span, particularly by extending into the coming years to capture the development trends of technological innovation over a broader range. This would not only provide more comprehensive data support but also enable a more accurate assessment of the dynamic relationships between population aging, technological innovation, and industrial development. Second, future research could delve into the data and conditions at the prefecture-level city to explore the relationship between population aging, artificial intelligence, and industrial differentiation across different regions. A more granular regional analysis would be more targeted and in-depth, presenting a more comprehensive picture of the variations in population aging, levels of technological innovation, and industrial differentiation in different areas, leading to conclusions that are more pertinent and persuasive.

## Data Availability

The raw data supporting the conclusions of this article will be made available by the authors, without undue reservation.
